# Healthcare Resource Utilization and Costs Related to Falls and Fractures Among People With Type 2 Diabetes Receiving Basal Insulin: The FRAGILE Study

**DOI:** 10.36469/001c.133274

**Published:** 2025-04-28

**Authors:** Guillermo E. Umpierrez, Elizabeth K. Pogge, Xuan Li, Ronald Preblick, Jasvinder Gill, Naushira Pandya

**Affiliations:** 1 Emory University School of Medicine, Department of Medicine, Atlanta, Georgia, USA; 2 Midwestern University College of Pharmacy, Glendale, Arizona, USA; 3 Sanofi, Bridgewater, New Jersey, USA; 4 Sanofi, Bridgewater, New Jersey, USA; 5 Kiran C. Patel College of Osteopathic Medicine, Nova Southeastern University, Fort Lauderdale, Florida, USA

**Keywords:** basal insulin, fracture, healthcare utilization, hypoglycemia, type 2 diabetes

## Abstract

**Background:**

The association between falls or fall-related fractures and hypoglycemia in people with type 2 diabetes is well established. Insulin treatment is associated with an increased risk of hypoglycemia, which is compounded in people of older age, but the risk is lower with longer-acting vs intermediate- or long-acting basal insulin analogs.

**Objective:**

To examine healthcare resource utilization and costs related to falls/fractures in people with type 2 diabetes treated with the longer-acting basal insulin Gla-300 (insulin glargine 300 U/mL) vs long-acting basal insulins (insulin glargine 100 U/mL or insulin detemir)/neutral protamine Hagedorn (NPH).

**Methods:**

This retrospective study of Optum’s de-identified Clinformatics® Data Mart Database compared data for people aged 50 years or older with at least 1 prescription claim for basal insulin (excluding insulin degludec) between April 1, 2015, and April 30, 2021, who initiated Gla-300 insulin (basal insulin–naive) or transitioned to Gla-300 from a different basal insulin (basal insulin–switch). Cohorts were propensity score–matched. The primary outcome was fall/fracture-related hospitalization and emergency department visit events (per 100 person-years of follow-up [P100PYFU]). The association between fall/fracture events and hypoglycemia and costs were secondary outcomes. Outcomes were compared using 95% confidence intervals of rate and other ratios; no statistical inference was performed.

**Results:**

Fall/fracture-related hospitalization (2.88 vs 3.33 P100PYFU) and emergency department visit events (5.28 vs 5.95 P100PYFU) were numerically lower in people who initiated basal insulin with Gla-300 vs long-acting basal insulins/NPH, and in those who switched to Gla-300 vs long-acting basal insulins/NPH (2.54 vs 3.38 and 4.48 vs 5.21 P100PYFU, respectively). People with vs without hypoglycemia experienced more falls/fractures, regardless of whether initiating basal insulin or switching basal insulin treatment. Costs tended to be lower for people who switched to Gla-300; however, low event rates caused variability.

**Conclusions:**

The results of this study suggest that there is a positive correlation between fall/fracture events and hypoglycemia in people with type 2 diabetes and also, that fall/fracture-related healthcare resource utilization was numerically lower in people who initiated basal insulin with Gla-300 vs long-acting basal insulins/NPH, and in those who switched to Gla-300 vs long-acting basal insulins/NPH.

## BACKGROUND

The US Centers for Disease Control and Prevention estimates that over 38 million Americans (approximately 1 in 10) have diabetes, with type 2 diabetes accounting for 90% to 95% of cases. About 1.2 million Americans aged 18 years or older were diagnosed with type 2 diabetes in 2021.^1^ The total estimated cost of diagnosed diabetes in the United States in 2022 was $412.9 billion, which included $306.6 billion in direct medical costs.^2^

Many factors can contribute to an increased risk of falls. Impaired balance and gait, polypharmacy, and history of previous falls have been identified as major risk factors; other risk factors include age, female gender, visual impairments, cognitive decline, reduction in lower extremity strength, cardiovascular disease, certain medications, depression, and environmental factors.^3^ Furthermore, results of a retrospective study of the National Health Interview Service database have revealed the importance of incorporating social determinants of health into fall risk assessments.^4^ Hypoglycemia has been associated with an increased risk of falls, both in community^5^ and hospital settings.^6,7^ The US Department of Health and Human Services identifies hypoglycemia as among the top 3 priorities for preventable adverse drug events.^8^ Older adults are at increased risk of hypoglycemia because of multiple comorbidities, attenuated counterregulatory mechanisms, polypharmacy, and hypoglycemia unawareness.^9^

Studies in people with type 2 diabetes have shown an association between hypoglycemia and fall-related fractures,^10–12^ along with other serious adverse events and mortality.^13^ The likelihood of falls is increased in those receiving insulin therapy, which itself is associated with an increased risk of hypoglycemia.^14^ A US public health surveillance study showed that older adults who received insulin were more than twice as likely as younger adults to visit the emergency department (ED) and nearly 5 times as likely to be hospitalized for insulin-related hypoglycemia.^15^

The most recent basal insulin analogs—insulin glargine 300 U/mL (Gla-300) and insulin degludec 100 and 200 U/mL (IDeg)—are termed longer-acting (second-generation) treatments because they have longer durations of action (>24 hours, and less variability) than the long-acting (first-generation) insulin glargine 100 U/mL (Gla-100).^16^

Data from randomized controlled trials demonstrate that Gla-300 has a lower hypoglycemia risk than intermediate-acting neutral protamine Hagedorn (NPH) insulin^17,18^ or the long-acting basal insulin analogs (Gla-100 and insulin detemir [IDet] 100 U/mL),^19–24^ with these findings being corroborated in real-world studies.^25–29^ The treat-to-target trial BEGIN Once Long^30^ compared treatment with IDeg-100 and Gla-100 in insulin-naive individuals with type 2 diabetes and revealed similar glycemic control and overall rates of confirmed hypoglycemia for IDeg and Gla-100, and fewer episodes of nocturnal confirmed hypoglycemia with IDeg. However, the association between hypoglycemia and risk of fall or fall-related fracture (fall/fracture) for longer-acting vs long-acting basal insulins/NPH has not been investigated. Results of the BRIGHT study^31^ (a head-to-head randomized controlled trial comparing Gla-300 and IDeg-100 insulin-naive patients with uncontrolled type 2 diabetes) revealed similar improvement in glycemic control and similar rates and incidence of hypoglycemia for Gla-300 and IDeg, with the exception of lower rates with Gla-300 in the titration period. Therefore, we used Gla-300 to represent the class of longer-acting basal insulin analogs.

This real-world, retrospective study evaluated fall/fracture-related healthcare resource utilization (HCRU) and costs in people with type 2 diabetes aged 50 years and older treated with longer-acting Gla-300 vs long-acting basal insulins (Gla-100/IDet 100 U/mL) or NPH insulin and also the association between fall/fracture events and hypoglycemia. As many organizations^32–35^ define older adults as those 65 years and older, the analysis was also conducted in the 65 years and older subgroup (see **Supplementary Online Material)**.

## METHODS

### Data Source

This study used secondary data from administrative claims in the Optum de-identified Clinformatics® Data Mart Database, which spans more than a decade and provides eligibility and enrollment information on over 77 million commercially insured and Medicare Advantage members in all 50 states.^36^

### Inclusion and Exclusion Criteria

**Figure 1.**
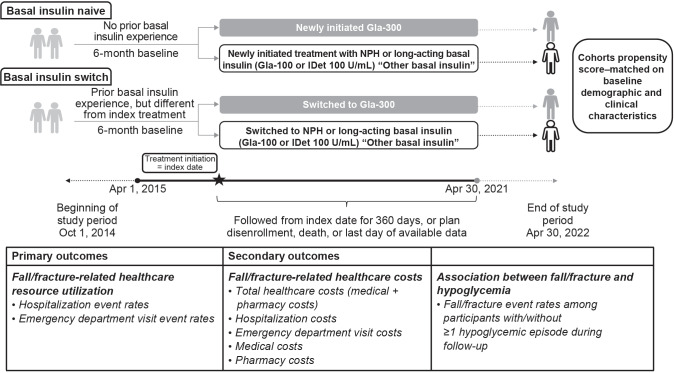
Study Design Abbreviations: Gla-100, insulin glargine 100 U/mL; Gla-300, insulin glargine 300 U/mL; IDet, insulin detemir; NPH, neutral protamine Hagedorn.

The cohort comprised people with a diagnosis of type 2 diabetes according to the *International Classification of Diseases* (ICD)-9 or ICD-10 who were aged 50 years and older and had at least 1 prescription claim for basal insulin (excluding insulin degludec [IDeg]) between April 1, 2015, and April 30, 2021 (**Figure 1**). Individuals with a diagnosis of other/type 1 diabetes at any time during the study period, those who had prescription claims for multiple basal insulins on the same day, and those who had prescription claims for IDeg during the study period were excluded. Participants were required to have at least 6 months of continuous enrollment prior to the basal insulin prescription fill. Follow-up ranged from 1 to 360 days.

### Study Groups and Exposure

Assessments were performed for both people with type 2 diabetes with no prior basal insulin use (basal insulin–naive cohort) and people who switched basal insulin from a different index basal insulin (basal insulin–switch cohort). Participants in the basal insulin–naive cohort had no previous basal insulin exposure in the 6 months (180 days) prior to baseline and newly initiated treatment with Gla-300, Gla-100, IDet, or NPH. Participants in the basal insulin–switch cohort had previously received Gla-100, IDet, or NPH and switched to either Gla-300 or a basal insulin different from their index basal insulin.

The basal insulin–switch cohort was identified using a hierarchical approach in which the first claim for Gla-300 was identified and participants were classified as switch users if they had a claim for a basal insulin other than Gla-300 during their baseline period. After these individuals were removed from the pool, the same process was repeated for Gla-100, IDet, and NPH, in that order. For each participant, only the first switch was considered. The primary analysis was conducted in the overall population aged 50 years and older.

### Identification of Falls/Fractures and Hypoglycemia

Falls/fractures were identified according to ICD-9-Clinical Modification (CM), ICD-9-Procedure Code Sets (PCS), ICD-10-CM, Current Procedural Terminology (CPT), and Healthcare Common Procedure Coding System (HCPCS) codes. Hypoglycemia was identified using either ICD-9-CM and ICD-10-CM codes, or a laboratory-confirmed fasting or non-fasting plasma glucose value of less than 70 mg/dL. Full details of codes are presented in **Table 1**.

**Table 1.** Fall/Fracture and Hypoglycemia Identification Codes

**Fall/Fracture Codes**
ICD-9-CM Codes
Falls: E880-E886, E888
Fractures [and dislocations]: E887, 800.X-829.X [Fracture codes] 830.X-839.X [Dislocation codes] 733.1X [Pathologic fracture] 733.93-733.98 [Stress fracture]
ICD-9-PCS codes
79.0-79.6 [Reduction of fracture procedure codes]
ICD-10-CM codes
Falls: W00.X-W19.X Slipping, tripping, stumbling and falls
Fractures: S02.X Fracture of skull and facial bones S03.0-S03.2 Dislocation of joints and ligaments of head S12.X Fracture of cervical vertebra and other parts of neck S13.0-S13.2 Dislocation of joints and ligaments at neck level S22.X Fracture of rib(s), sternum and thoracic spine S23.0-S23.2 Dislocation of joints and ligaments of thorax S32.X Fracture of lumbar spine and pelvis S33.0-S33.3 Dislocation of joints and ligaments of lumbar spine and pelvis S42.X Fracture of shoulder and upper arm S43.0-S43.3 Dislocation of joints and ligaments of shoulder girdle S52.X Fracture of forearm S53.0-S53.3 Dislocation of joints and ligaments of elbow S62.X Fracture at wrist and hand level S63.0-S63.4 Dislocation of joints and ligaments at wrist and hand level S72.X Fracture of femur S73.0 Dislocation of joint and ligaments of hip S82.X Fracture of lower leg, including ankle S83.0-S83.3 Dislocation of joints and ligaments of knee S92.X Fracture of foot and toe, except ankle S93.0-S93.3 Dislocation of joints and ligaments at ankle, foot and toe level M80.X Osteoporosis with current pathological fracture M84.3X Stress fracture M84.4X Pathological fracture, not elsewhere classified M84.5X Pathological fracture in neoplastic disease M84.6X Pathological fracture in other disease M84.7X Nontraumatic fracture, not elsewhere classified
CPT codes
21800, 21805, 21810, 21820, 21825, 22305, 22310, 22318, 22319, 22520, 22521, 22523, 22524, 23500, 23505, 23515, 23570, 23575, 23585, 23600, 23605, 23615, 23616, 23620, 23625, 23630, 23665, 23670, 23675, 23680, 24500, 24505, 24515, 24516, 24530, 24535, 24538, 24545, 24546, 24560, 24565, 24566, 24575, 24576, 24577, 24579, 24582, 24620, 24635, 24650, 24655, 24665, 24666, 24670, 24675, 24685, 25500, 25505, 25515, 25520, 25525, 25526, 25530, 25535, 25545, 25560, 25565, 25574, 25575, 25600, 25605, 25606, 25607, 25608, 25609, 25622, 25624, 25628, 25630, 25635, 25645, 25650, 25651, 25652, 25680, 25685, 26600, 26605, 26607, 26608, 26615, 27193, 27194, 27200, 27202, 27215, 27216, 27217, 27218, 27220, 27222, 27226, 27227, 27228, 27230, 27232, 27235, 27236, 27238, 27240, 27244, 27245, 27246, 27248, 27254, 27267, 27268, 27269, 27500, 27501, 27502, 27503, 27506, 27507, 27508, 27509, 27510, 27511, 27513, 27514, 27520, 27524, 27530, 27532, 27535, 27536, 27538, 27540, 27750, 27752, 27756, 27758, 27759, 27760, 27762, 27766, 27767, 27768, 27769, 27780, 27781, 27784, 27786, 27788, 27792, 27808, 27810, 27814, 27816, 27818, 27822, 27823, 27824, 27825, 27826, 27827, 27828, 28400, 28405, 28406, 28415, 28420, 28430, 28435, 28436, 28445, 28450, 28455, 28456, 28465, 28470, 28475, 28476, 28485, 29850, 29851, 29855, 29856
HCPCS code: S2360
**Hypoglycemia Codes**
ICD-9-CM codes
251.0, 251.1, 251.2, 270.3 and 250.8 (not with 259.8, 272.7, 523.8/523.9, 681-682, 692.9, 707.1-707.9, 709.3, 730.0–730.2, 731.8)
ICD-10-CM codes
E0864, E08641, E08649, E0964, E09641, E09649, E1064, E10641, E10649, E1164, E11641, E11649, E1364, E13641, E13649, E15, E160, E161, E162

Abbreviations: HCPCS, Healthcare Common Procedure Coding System; ICD, *International Classification of Disease*.

### Propensity Score Matching

Cohorts were 1:1 propensity score–matched according to baseline demographic and clinical characteristics using a greedy nearest-neighbor matching algorithm and a caliper of 0.2 SD of the logit of the propensity score. Once a match was made, participants were not reconsidered. A standardized mean difference less than 0.1 indicated balance between groups with any remaining imbalanced characteristics accounted for by inclusion as covariates in outcome models.

The following demographic and baseline characteristics (determined by ICD-9 or ICD-10 codes) were considered to be associated with risk of fall/fracture and were included in the propensity score–matching (PSM) model: age, gender, healthcare plan type, region, baseline comorbidities/complications, baseline use of oral or injectable antidiabetic agents, diabetes polypharmacy (intake of ≥3 different noninsulin antidiabetic agents within 30 days of index date), history of hypoglycemia, history of falls/fractures, frailty and age-related factors (vision loss and impairment, cognitive impairment and dementia, age-related physical debility, osteoarthritis, osteoporosis), medications (antipsychotics, antidepressants, benzodiazepines, polypharmacy), high-risk triggering events and conditions (hypotension/postural hypotension, vertigo, syncope and collapse, nonepileptic seizure, malaise, fatigue), and baseline glycated hemoglobin A1C. Demographic and baseline characteristics before and after PSM are detailed in **Table 2**.

**Table 2.** Demographic and Baseline Characteristics in People With Type 2 Diabetes Aged ≥50 Years Before and After PSM

**Demographic and Clinical Characteristics**	**Basal Insulin–Naive Population**						**Basal Insulin–Switch Population**					
	**Before PSM**			**After PSM**			**Before PSM**			**After PSM**		
	**Gla-300 (n = 14 534)**	**Other Long- Acting BIs (n = 137 332)**	**SMD**	**Gla-300 (n = 14 533)**	**Other Long-Acting BIs (n = 14 533)**	**SMD**	**Gla-300 (n = 10 066)**	**Other Long-Acting BIs (n = 13 608)**	**SMD**	**Gla-300 (n = 8893)**	**Other Long-Acting BIs (n = 8893)**	**SMD**
Age (years), continuous												
Mean (SD)	68.69 (8.70)	67.73 (9.90)	0.10	68.69 (8.70)	68.52 (8.91)	0.02	66.49 (8.85)	66.07 (9.79)	0.04	66.30 (8.92)	66.07 (9.37)	0.02
Median (IQR)	69.0 (63.0-75.0)	68.0 (60.0-75.0)		69.0 (63.0-75.0)	69.0 (63.0-74.0)		67.0 (60.0-72.0)	65.0 (58.0-73.0)		66.0 (59.0-72.0)	66.0 (59.0-72.0)	
Minimum (maximum)	50.0 (90.0)	50.0 (90.0)		50.0 (90.0)	50.0 (90.0)		50.0 (90.0)	50.0 (90.0)		50.0 (90.0)	50.0 (90.0)	
Gender, n (%)												
Male	7309 (50.3)	71 630 (52.2)	0.04	7309 (50.3)	7274 (50.1)	0.00	5062 (50.3)	6743 (49.6)	0.01	4504 (50.6)	4495 (50.5)	0.00
Female	7225 (49.7)	65 702 (47.8)		7224 (49.7)	7259 (49.9)		5004 (49.7)	6865 (50.4)		4389 (49.4)	4398 (49.5)	
Health plan type, n (%)												
Commercial	2299 (15.8)	42 228 (30.7)	0.36	2299 (15.8)	2332 (16.0)	0.01	3328 (33.1)	6451 (47.4)	0.30	3230 (36.3)	3688 (41.5)	0.11
Medicare	12 231 (84.2)	95 070 (69.2)		12 230 (84.2)	12 196 (83.9)		6735 (66.9)	7155 (52.6)		5660 (63.6)	5203 (58.5)	
Region, n (%)												
Northeast	1127 (7.8)	12 858 (9.4)	0.55	1127 (7.8)	1223 (8.4)	0.46	842 (8.4)	1230 (9.0)	0.18	730 (8.2)	763 (8.6)	0.09
Midwest	1990 (13.7)	29 372 (21.4)	0.41	1990 (13.7)	2506 (17.2)	0.40	1901 (18.9)	3155 (23.2)	0.08	1737 (19.5)	1871 (21.0)	0.06
South	8841 (60.8)	65 924 (48.0)	0.21	8840 (60.8)	7356 (50.6)	0.16	5396 (53.6)	6852 (50.4)	0.13	4766 (53.6)	4588 (51.6)	0.04
West	2564 (17.6)	28 998 (21.1)	0.49	2564 (17.6)	3431 (23.6)	0.32	1915 (19.0)	2356 (17.3)	0.19	1649 (18.5)	1661 (18.7)	0.09
Other/unknown	12 (0.1)	180 (0.1)	0.57	12 (0.1)	17 (0.1)	0.47	12 (0.1)	15 (0.1)	0.19	11 (0.1)	10 (0.1)	0.09
Index basal insulin, n (%)												
Gla-100	N/A	101 312 (73.8)	N/A	N/A	10 775 (74.1)	N/A	N/A	12 539 (92.1)	N/A	N/A	8202 (92.2)	N/A
Insulin detemir		26 884 (19.6)			2758 (19.0)			973 (7.2)			614 (6.9)	
NPH		9136 (6.7)			1000 (6.9)			96 (0.7)			77 (0.9)	
Baseline comorbidities and complications, n (%)												
Anemia	2447 (16.8)	28 576 (20.8)	-0.10	2447 (16.8)	2376 (16.3)	0.01	1497 (14.9)	2847 (20.9)	-0.16	1355 (15.2)	1377 (15.5)	-0.01
Hypertension	11 680 (80.4)	108 884 (79.3)	0.03	11 679 (80.4)	11 645 (80.1)	0.01	8147 (80.9)	11 115 (81.7)	-0.02	7214 (81.1)	7169 (80.6)	0.01
Hyperlipidemia	9571 (65.9)	79 666 (58.0)	0.16	9570 (65.9)	9509 (65.4)	0.01	6678 (66.3)	8641 (63.5)	0.06	5880 (66.1)	5693 (64.0)	0.04
Diabetic neuropathy	4304 (29.6)	39 377 (28.7)	0.02	4303 (29.6)	4264 (29.3)	0.01	3415 (33.9)	4272 (31.4)	0.05	2956 (33.2)	2781 (31.3)	0.04
Diabetic nephropathy	1305 (9.0)	11 403 (8.3)	0.02	1305 (9.0)	1251 (8.6)	0.01	984 (9.8)	1257 (9.2)	0.02	866 (9.7)	816 (9.2)	0.02
Diabetic retinopathy	1564 (10.8)	11 761 (8.6)	0.07	1564 (10.8)	1453 (10.0)	0.03	1480 (14.7)	1761 (12.9)	0.05	1275 (14.3)	1193 (13.4)	0.03
Overweight/obesity	3313 (22.8)	32 637 (23.8)	-0.02	3313 (22.8)	3250 (22.4)	0.01	2741 (27.2)	3713 (27.3)	0.00	2425 (27.3)	2350 (26.4)	0.02
Chronic kidney disease	4005 (27.6)	35 502 (25.9)	0.04	4004 (27.6)	3890 (26.8)	0.02	2534 (25.2)	3655 (26.9)	-0.04	2238 (25.2)	2144 (24.1)	0.02
Depression	2170 (14.9)	22 818 (16.6)	-0.05	2170 (14.9)	2106 (14.5)	0.01	1526 (15.2)	2468 (18.1)	-0.08	1351 (15.2)	1367 (15.4)	-0.01
Anxiety	1488 (10.2)	16 820 (12.2)	-0.06	1488 (10.2)	1464 (10.1)	0.01	1091 (10.8)	1739 (12.8)	-0.06	987 (11.1)	977 (11.0)	0.00
Ischemic heart disease	3407 (23.4)	35 903 (26.1)	-0.06	3407 (23.4)	3280 (22.6)	0.02	2390 (23.7)	3516 (25.8)	-0.05	2134 (24.0)	2063 (23.2)	0.02
Myocardial infarction	251 (1.7)	5823 (4.2)	-0.15	251 (1.7)	241 (1.7)	0.01	169 (1.7)	547 (4.0)	-0.14	164 (1.8)	166 (1.9)	0.00
Transient ischemic attack	203 (1.4)	3035 (2.2)	-0.06	203 (1.4)	186 (1.3)	0.01	140 (1.4)	305 (2.2)	-0.06	131 (1.5)	136 (1.5)	0.00
Stroke	419 (2.9)	7450 (5.4)	-0.13	419 (2.9)	361 (2.5)	0.02	265 (2.6)	827 (6.1)	-0.17	256 (2.9)	249 (2.8)	0.00
Peripheral arterial disease	627 (4.3)	5416 (3.9)	0.02	626 (4.3)	581 (4.0)	0.02	358 (3.6)	620 (4.6)	-0.05	324 (3.6)	327 (3.7)	0.00
Heart failure	1782 (12.3)	23 870 (17.4)	-0.14	1782 (12.3)	1679 (11.6)	0.02	1250 (12.4)	2444 (18.0)	-0.15	1153 (13.0)	1139 (12.8)	0.00
Baseline oral and injectable antidiabetics												
Diabetes polypharmacy^a^	557 (3.8)	4795 (3.5)	0.02	557 (3.8)	535 (3.7)	0.01	366 (3.6)	373 (2.7)	0.05	311 (3.5)	318 (3.6)	0.00
Prior insulin^b^												
Switched from Gla-100	N/A	N/A	N/A	N/A	N/A	N/A	7047 (70.0)	286 (2.1)	N/A	6271 (70.5)	186 (2.1)	N/A
Switched from IDet							2450 (24.3)	9727 (71.5)		2122 (23.9)	6264 (70.4)	
Switched from NPH							569 (5.7)	3595 (26.4)		500 (5.6)	2443 (27.5)	
Biguanides	7268 (50.0)	65 659 (47.8)	0.04	7268 (50.0)	7304 (50.3)	0.00	4902 (48.7)	6559 (48.2)	0.01	4358 (49.0)	4368 (49.1)	0.00
Sulfonylureas	5581 (38.4)	49 343 (35.9)	0.05	5581 (38.4)	5487 (37.8)	0.01	2380 (23.6)	3212 (23.6)	0.00	2103 (23.6)	2123 (23.9)	-0.01
Thiazolidindiones	1209 (8.3)	8669 (6.3)	0.08	1209 (8.3)	1162 (8.0)	0.01	547 (5.4)	643 (4.7)	0.03	480 (5.4)	465 (5.2)	0.01
DPP-4 inhibitors	2361 (16.2)	18 776 (13.7)	0.07	2361 (16.2)	2305 (15.9)	0.01	1125 (11.2)	1360 (10.0)	0.04	1001 (11.3)	945 (10.6)	0.02
SGLT2 inhibitors	1679 (11.6)	9926 (7.2)	0.15	1678 (11.5)	1601 (11.0)	0.02	1336 (13.3)	1279 (9.4)	0.12	1143 (12.9)	1137 (12.8)	0.00
GLP-1 RA	2462 (16.9)	13 757 (10.0)	0.20	2461 (16.9)	2361 (16.2)	0.02	2346 (23.3)	1932 (14.2)	0.23	1871 (21.0)	1836 (20.6)	0.01
α-Glucosidase inhibitors	81 (0.6)	646 (0.5)	0.01	81 (0.6)	75 (0.5)	0.01	37 (0.4)	29 (0.2)	0.03	33 (0.4)	27 (0.3)	0.01
Other hypoglycemic agents	226 (1.6)	1553 (1.1)	0.04	225 (1.5)	204 (1.4)	0.01	117 (1.2)	114 (0.8)	0.03	98 (1.1)	95 (1.1)	0.00
Combination formulations	1241 (8.5)	7615 (5.5)	0.12	1240 (8.5)	1215 (8.4)	0.01	792 (7.9)	783 (5.8)	0.08	690 (7.8)	668 (7.5)	0.01
History of hypoglycemia												
Any hypoglycemia during baseline (yes/no)	430 (3.0)	5555 (4.0)	-0.06	430 (3.0)	384 (2.6)	0.02	431 (4.3)	1009 (7.4)	-0.13	394 (4.4)	422 (4.7)	-0.02
History of fall/fractures												
Any fall/fractures during baseline (yes/no)	610 (4.2)	9394 (6.8)	-0.12	610 (4.2)	591 (4.1)	0.01	410 (4.1)	1065 (7.8)	-0.16	386 (4.3)	418 (4.7)	-0.02
No. of OADs (continuous)												
Mean (SD)	1.52 (1.12)	1.28 (1.10)	0.22	1.52 (1.12)	1.49 (1.16)	0.02	1.35 (1.03)	1.17 (1.01)	0.18	1.32 (1.02)	1.31 (1.05)	0.01
Median (IQR)	1.0 (1.0-2.0)	1.0 (0.0-2.0)	N/A	1.0 (1.0-2.0)	1.0 (1.0-2.0)	N/A	1.0 (1.0-2.0)	1.0 (0.0-2.0)	N/A	1.0 (1.0-2.0)	1.0 (0.0-2.0)	–
Minimum (maximum)	0.0 (6.0)	0.0 (7.0)	N/A	0.0 (6.0)	0.0 (7.0)	N/A	0.0 (6.0)	0.0 (7.0)	N/A	0.0 (6.0)	0.0 (7.0)	–
No. of OADs (categorical), n (%)												
0	2967 (20.4)	40 485 (29.5)	0.15	2967 (20.4)	3394 (23.4)	0.06	2253 (22.4)	4012 (29.5)	0.13	2029 (22.8)	2234 (25.1)	0.07
1	4506 (31.0)	41 870 (30.5)	0.31	4506 (31.0)	4241 (29.2)	0.10	3677 (36.5)	4918 (36.1)	0.25	3306 (37.2)	3083 (34.7)	0.07
2	4308 (29.6)	35 418 (25.8)	0.29	4308 (29.6)	4054 (27.9)	0.10	2805 (27.9)	3292 (24.2)	0.22	2441 (27.4)	2384 (26.8)	0.10
≥3	2753 (18.9)	19 559 (14.2)	0.28	2752 (18.9)	2844 (19.6)	0.11	1331 (13.2)	1386 (10.2)	0.22	1117 (12.6)	1192 (13.4)	0.10
Risk factors for falls/fractures												
Frailty and age-related factors, n (%)												
Vision loss and impairment^c^	120 (0.8)	1509 (1.1)	-0.03	120 (0.8)	111 (0.8)	0.01	81 (0.8)	182 (1.3)	-0.05	75 (0.8)	68 (0.8)	0.01
Cognitive impairment and dementia	502 (3.5)	10 070 (7.3)	-0.17	502 (3.5)	511 (3.5)	0.00	339 (3.4)	1178 (8.7)	-0.22	334 (3.8)	378 (4.3)	-0.03
Age-related physical debility	37 (0.3)	1046 (0.8)	-0.07	37 (0.3)	30 (0.2)	0.01	25 (0.2)	132 (1.0)	-0.09	25 (0.3)	29 (0.3)	-0.01
Osteoarthritis	2633 (18.1)	26 106 (19.0)	-0.02	2633 (18.1)	2589 (17.8)	0.01	1802 (17.9)	2654 (19.5)	-0.04	1589 (17.9)	1555 (17.5)	0.01
Osteoporosis	584 (4.0)	4812 (3.5)	0.03	584 (4.0)	573 (3.9)	0.00	315 (3.1)	473 (3.5)	-0.02	279 (3.1)	266 (3.0)	0.01
Medications												
Antipsychotics	558 (3.8)	6832 (5.0)	-0.06	558 (3.8)	582 (4.0)	-0.01	428 (4.3)	695 (5.1)	-0.04	385 (4.3)	375 (4.2)	0.01
Antidepressants	4351 (29.9)	39 160 (28.5)	0.03	4351 (29.9)	4280 (29.5)	0.01	3344 (33.2)	4425 (32.5)	0.01	2938 (33.0)	2862 (32.2)	0.02
Benzodiazepines	172 (1.2)	1347 (1.0)	0.02	172 (1.2)	163 (1.1)	0.01	116 (1.2)	119 (0.9)	0.03	108 (1.2)	89 (1.0)	0.02
Polypharmacy^d^	5944 (40.9)	54 745 (39.9)	0.02	5944 (40.9)	5896 (40.6)	0.01	5069 (50.4)	6735 (49.5)	0.02	4450 (50.0)	4428 (49.8)	0.00
High-risk triggering events and conditions, n (%)												
Hypotension and postural hypotension	395 (2.7)	7522 (5.5)	-0.14	395 (2.7)	353 (2.4)	0.02	236 (2.3)	719 (5.3)	-0.15	225 (2.5)	237 (2.7)	-0.01
Vertigo	149 (1.0)	1182 (0.9)	0.02	149 (1.0)	113 (0.8)	0.03	88 (0.9)	114 (0.8)	0.00	77 (0.9)	77 (0.9)	0.00
Syncope and collapse	417 (2.9)	5808 (4.2)	-0.07	417 (2.9)	410 (2.8)	0.00	254 (2.5)	608 (4.5)	-0.11	232 (2.6)	268 (3.0)	-0.02
Nonepileptic seizure	114 (0.8)	2621 (1.9)	-0.10	114 (0.8)	109 (0.8)	0.00	102 (1.0)	299 (2.2)	-0.09	95 (1.1)	103 (1.2)	-0.01
Malaise and fatigue	2256 (15.5)	28 852 (21.0)	-0.14	2256 (15.5)	2138 (14.7)	0.02	1360 (13.5)	2823 (20.7)	-0.19	1255 (14.1)	1267 (14.2)	0.00
Healthcare utilization, n (%)												
≥1 inpatient admission	1749 (12.0)	40 695 (29.6)	-0.44	1749 (12.0)	1625 (11.2)	0.03	1118 (11.1)	3561 (26.2)	-0.39	1107 (12.4)	1176 (13.2)	-0.02
≥1 ED visit	3342 (23.0)	50 418 (36.7)	-0.30	3342 (23.0)	3173 (21.8)	0.03	2129 (21.2)	4501 (33.1)	-0.27	1973 (22.2)	2064 (23.2)	-0.02

Abbreviations: DDP4, dipeptidyl peptidase 4; ED, emergency department; GLP-1 RA, glucagon-like peptide 1 receptor agonists; IDet, insulin detemir; Gla-100, insulin glargine 100 U/mL; Gla-300, insulin glargine 300 U/mL; IQR, interquartile range; NA, not available; NPH, neutral protamine Hagedorn; OADs, oral antidiabetic drugs; PSM, propensity score–matching; SGLT2, sodium-glucose cotransporter-2; SMD, standardized mean difference.

^a^At least 3 different noninsulin antidiabetics within 30 days of index.

^b^SMD for prior insulin is N/A because prior insulin was not included as a covariate in the PSM model.

^c^Vision loss and impairment beyond diabetic ophthalmopathy (eg, cataracts).

^d^At least 5 different nondiabetes medications within 30 days of index.

After matching, baseline characteristics were considered balanced between cohorts, with any imbalances that persisted after PSM included as covariates in analysis models. Baseline sulfonylurea use, HCRU, and history of falls and hypoglycemia were similar between treatment groups for both age groups and for both the basal insulin–naive and basal insulin–switch cohorts. The basal insulin–naive model (n = 14 533/group) was adjusted for region and number of oral antidiabetic drugs, while the basal insulin–switch model (n = 8893/group) was adjusted for healthcare plan type.

### Study Outcomes

The primary outcome was HCRU (hospitalizations and ED visits) related to falls or fractures. Secondary outcomes were investigation of the crude association between fall/fracture events and hypoglycemia (assessed by fall/fracture event rates among participants with vs without at least 1 hypoglycemic episode during follow-up) as well as assessment of fall/fracture-related paid costs (hospitalization, ED visit, and total health care [all medical and pharmacy costs]). Medical costs included outpatient, inpatient, ED, laboratory, imaging, and other costs, excluding pharmacy costs.

### Data Analysis

Results were reported as 95% confidence intervals for rate and other ratios; *P* values and statistical inference were not reported.

## RESULTS

### Demographic and Baseline Characteristics

Between April 1, 2015, and April 30, 2021, 95 564 and 838 996 people with type 2 diabetes were identified with at least 1 prescription claim(s) for Gla-300 or long-acting basal insulins/NPH, respectively. For the basal insulin–naive cohort, 14 534 participants who received Gla-300 and 137 332 who received long-acting basal insulins/NPH were included in the analysis. The basal insulin–switch cohort consisted of 10 066 participants who received Gla-300 and 13 608 who received long-acting basal insulins/NPH (**Supplementary Table S1**).

### Primary Outcome: Fall/Fracture-Related HCRU

**Figure 2.**
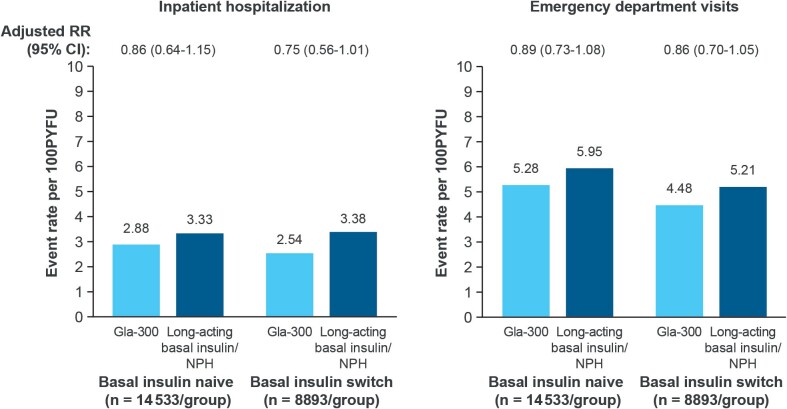
Fall/Fracture-Related Healthcare Resource Utilization in People With Type 2 Diabetes Aged ≥50 Years Abbreviations: CI, confidence interval; Gla-300, insulin glargine 300 U/mL; NPH, neutral protamine Hagedorn; PYFU, person-years of follow-up; RR, rate ratio.

For the primary outcome in the basal insulin–naive cohort, following PSM, both fall/fracture-related hospitalization (2.88 vs 3.33 events per 100 person-years of follow-up [P100PYFU]) and ED visit event rates (5.28 vs 5.95 events P100PYFU) were numerically lower in people who initiated Gla-300 vs long-acting basal insulins/NPH. Results were similar in the basal insulin–switch cohort, with those who switched to Gla-300 having numerically lower hospitalizations (2.54 vs 3.38 events) and ED visits (4.48 vs 5.21 events; **Figure 2**).

### Association Between Falls/Fractures and Hypoglycemia

**Figure 3.**
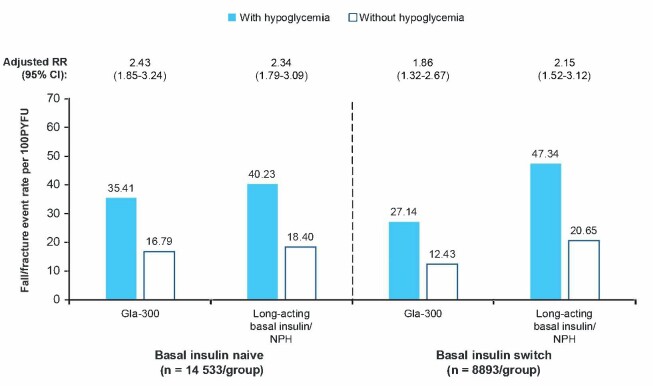
Association Between Fall/Fracture and Hypoglycemia in People With Type 2 Diabetes Aged ≥50 Years Abbreviations: CI, confidence interval; ED, emergency department; Gla-300, insulin glargine 300U/mL; NPH, neutral protamine Hagedorn; PYFU, person-years of follow-up; RR, rate ratio.

The results of this retrospective study suggest a positive association between falls/fractures and the incidence of hypoglycemia. Fall/fracture event rates were numerically higher for people with vs without hypoglycemia, regardless of whether participants were newly initiating basal insulin or switching basal insulin (**Figure 3**). There were numerically fewer falls/fractures in people who received Gla-300 vs long-acting basal insulins/NPH across all comparisons, with the difference being particularly notable in the basal insulin–switch cohort.

### Fall/Fracture-Related Healthcare Paid Costs

In the basal insulin–naive cohort, fall/fracture-related total healthcare costs (P100PYFU) were numerically higher for people who initiated Gla-300 vs long-acting basal insulins/NPH ($111 091 vs $106 833, respectively). Total healthcare costs consisted of medical costs (which were also numerically higher for Gla-300 vs long-acting basal insulins [$110 934 vs $106 620]) and pharmacy costs (which were numerically lower for Gla-300 [$156 vs $213]). Emergency department visit costs were numerically higher for people who initiated Gla-300 vs long-acting basal insulins/NPH ($23 382 vs $20 938), but hospitalization costs were similar between the groups ($73 958 vs $73 884) (**Table 3**). In the basal insulin–switch cohort, fall/fracture-related total healthcare costs were substantially lower for Gla-300 ($78 929 vs $124 356, respectively), with most of the cost saving being due to reduced medical costs ($78 595 vs $124 045), as pharmacy costs ($334 vs $310) were similar. Hospitalization costs were also substantially lower for people who switched to Gla-300 vs long-acting basal insulins/NPH ($53 927 vs $93 077), whereas ED visit costs ($16 845 vs $16 821) were similar (**Table 3**).

**Table 3.** Fall/Fracture-Related Costs After PSM

**Fall/Fracture-Related Cost, $ per 100 PYFU^a^**	**People With Type 2 Diabetes Aged ≥50 y**							
	**Basal Insulin–Naive Population (n = 14 533)**				**Basal Insulin–Switch Population (n = 8893)**			
	**Gla-300**	**Long-Acting BIs/NPH**	**Cost Difference (Gla-300 vs Long-Acting BIs/NPH)**	**Adjusted Cost Ratio (95% CI): Gla-300 vs Long-Acting BIs/NPH**	**Gla-300**	**Long-Acting BIs/NPH**	**Cost Difference (Gla-300 vs Long-Acting BIs/NPH)**	**Adjusted Cost Ratio (95% CI): Gla-300 vs Long-Acting BIs/NPH**
Hospitalization	73 958 (n = 295)	73 884 (n = 330)	+74	0.97 (0.72-1.31)	53 927 (n = 160)	93 077 (n = 195)	-39 150	0.48 (0.33-0.71)
ED visit	23 382 (n = 589)	20 938 (n = 631)	+2444	1.17 (1.02-1.35)	16 845 (n = 308)	16 821 (n = 342)	+24	1.08 (0.90-1.30)
Total health care	111 091 (n = 835)	106 833 (n = 924)	+4258	0.69 (0.49-0.97)	78 929 (n = 449)	124 356 (n = 518)	-45 426	0.47 (0.37-0.62)
Medical	110 934	106 620	+4314	0.69 (0.49-0.97)	78 595	124 045	-45 450	0.49 (0.38-0.63)
Pharmacy	156	213	-56	1.23 (0.47-3.23)	334	310	+24	1.69 (0.65-4.40)

Abbreviations: BI, basal insulin; CI, confidence interval; Gla-300, insulin glargine 300 U/mL; NPH, neutral protamine Hagedorn; PSM, propensity score–matching; PYFU, person-years of follow-up.

^a^Participants with ≥1 event.

It is important to note that absolute cost differences between Gla-300 and long-acting basal insulins/NPH did not consistently fall in the same direction as the adjusted cost ratio.

## DISCUSSION

The incidence of falls/fractures among adults is increasing,^37^ with risk being higher in those with diabetes.^38^ However, hypoglycemia rates are lower for longer-acting vs intermediate- or long-acting basal insulins.^17–28^ As such, this retrospective study aimed to assess fall/fracture-related outcomes in people with type 2 diabetes aged 50 years and older treated with the longer-acting basal insulin Gla-300 vs the long-acting basal insulins Gla-100 or IDet 100 U/mL, or NPH insulin. The results revealed that fall/fracture-related hospitalization and ED visit event rates in people with type 2 diabetes aged 50 years and older were numerically lower in participants who received Gla-300 vs long-acting basal insulins or NPH insulin, regardless of whether they were naive to basal insulin or switched from another basal insulin.

Our study findings also suggest a positive association between fall/fracture events and hypoglycemia, with incidence rates being numerically higher in individuals with vs without hypoglycemia in all comparisons. However, fall/fracture rates in people with hypoglycemia were variable, and it is notable that rates were lowest in those who switched to Gla-300. Regardless, there were numerically fewer falls/fractures in people who received Gla-300 vs long-acting basal insulins/NPH in both the treatment-naive and the treatment-switch settings.

The HCRU findings in the basal insulin–naive and basal insulin–switch populations corroborate those from other studies. In DELIVER Naive, in people newly initiating basal insulin, rates of any hypoglycemia were similar for Gla-300 and Gla-100, and although rates of hypoglycemia associated with inpatient/ED visits were significantly lower for Gla-300 vs Gla-100 at 3 months (0.04 vs 0.17 per person per year; least squares mean difference, -0.13; *P* = .003), the difference at 6 months was not significant (0.09 vs 0.15; least squares mean difference, -0.07; *P* = .093).^39^ Similar to the current study, the DELIVER 2 and DELIVER 3 studies^25,26^ reported that in people with type 2 diabetes who switched basal insulin, rates of both hypoglycemia and hypoglycemia associated with inpatient/ED visits were significantly lower in people who switched to Gla-300 vs Gla-100 or IDet 100 U/mL.

In the basal insulin–naive cohorts, the observed mean fall/fracture-related cost differences P100PY were mostly numerically higher for people initiating Gla-300 vs long-acting basal insulins/NPH. This increase was largely driven by increased medical and ED visit costs, with hospitalization costs being similar between treatment groups. However, in the population who switched from another basal insulin, most fall/fracture-related costs were substantially lower for people switching to Gla-300 vs long-acting basal insulins/NPH, except for similar ED visit and pharmacy costs. These results are similar to those of the DELIVER 2 real-world analysis, which showed that switching to Gla-300 vs another basal insulin was associated with an overall HCRU saving of $1439 per person per year.^25^

It is noteworthy that although costs for Gla-300 decreased in the basal insulin–switch setting relative to the basal insulin–naive setting, costs related to long-acting basal insulins/NPH increased, thus enhancing the apparent cost savings for Gla-300. The finding that most pharmacy costs were numerically higher for Gla-300 than for long-acting basal insulin/NPH analogs is similar to that reported in another claims-based analysis, which showed that for people with type 2 diabetes initially receiving long-acting basal insulin/NPH, switching to Gla-300 vs Gla-100 or IDet 100 U/mL was associated with similar total healthcare costs despite higher pharmacy costs.^40^ As shown in that study, increased pharmacy costs with Gla-300 may be reflective of better treatment persistence with Gla-300 vs long-acting basal insulin analogs/NPH.^40^

### Strengths and Limitations

The main strength of this analysis is that Optum’s de-identified Clinformatics® Data Mart Database has wide subject coverage and availability of criteria (eg, exposure, outcomes). However, the database may not be representative of all US-based healthcare plan policies; further, it captures only administrative claims submitted for payment by providers and pharmacies, and as such may miss data from individuals paying out of pocket. The study was retrospective, and as no *P* values or statistical inference was performed, the results are hypothesis-generating only. Other limitations include those common to all administrative claims database analyses (eg, possible coding errors, including medical diagnoses or hypoglycemia). Although the accuracy of major diagnosis codes from the claims database is considered acceptable, the validity of falls and fracture codes might still be considered a limitation. Additionally, while claims data denote the date of prescription fills and days’ supply, information on actual use of medications is not available; it is possible that basal insulin therapy was discontinued after the index date, and differing rates of treatment adherence and discontinuation could have biased the results. Confounding from unmeasured or incorrectly specified confounders may have occurred; however, PSM should have reduced the likelihood of this by ensuring that the distribution of the measured covariates was balanced. Nevertheless, it is notable that there were fewer switches from NPH insulin to Gla-300 than to Gla-100 or IDet. As NPH insulin has the highest associated hypoglycemia risk of all investigated basal insulins, this could have contributed to an underestimation of the effect of switching to Gla-300.

An important limitation of this study is that the low number of events limited interpretation of costs data. Absolute cost differences between Gla-300 and NPH/long-acting basal insulins did not consistently fall in the same direction as the adjusted cost ratio. The event rates in the study were low (~2%), and as cost differences were calculated using mean data, the mean value could have been skewed by data for some people with particular circumstances leading to increased costs. For example, one study reported that medical and hospital-related costs vary depending on the hospital.^41^ Furthermore, findings of meta-analysis and systematic reviews also suggest that the direction of association between healthcare cost and quality can be inconsistent.^42,43^ The present study did not include insulin degludec. However, because data from randomized controlled trials confirm that the 2 longer-acting basal insulin analogs (Gla-300 and IDeg) have comparable glycemic efficacy,^31^ it is likely that the results of this study can be extrapolated.

## CONCLUSIONS

The results of this retrospective study indicate that Gla-300 is associated with numerically lower fall/fracture-related hospitalization and ED visit event rates compared with long-acting basal insulins (Gla-100, IDet 100 U/mL) or NPH insulin, regardless of whether individuals were initiating Gla-300 or switching to Gla-300 from a different basal insulin. However, cost analysis suggests that this benefit does not necessarily result in cost savings.

### Disclosures

G.E.U. has received research grants from the National Institutes of Health (NIH/NCATS UL 3UL1TR002378-05S2), the Clinical and Translational Science Award program, and the National Institutes of Health and National Center for Research Resources (NIH/NIDDK 2P30DK111024-06); has received research support (to Emory University) from Abbott, Bayer, and Dexcom; and has served on advisory boards for Dexcom and GlyCare Health. E.K.P. has received consultancy fees from Sanofi. X.L. was an employee of Sanofi at the time of study conduct. R.P. and J.G. are employees of Sanofi and may hold stocks/shares in Sanofi. N.P. has received funding from the Health Resources and Services Administration (HRSA 6 U1QHP33074-05-01) for the Geriatrics Workforce Enhancement Program (GWEP).

### Contributions

X.L., R.P., and J.G. contributed to the study design and acquisition and analyses of the data. All authors were involved in the interpretation of the data and in drafting and revising the manuscript for important intellectual content, and all approved the publication for submission and agreed to be accountable for all aspects of the work in ensuring that questions related to the accuracy or integrity of any part of the work are appropriately investigated and resolved. G.E.U. is the guarantor of this work, and as such, had full access to all the data in the study and takes responsibility for the overall integrity of the article.

## Supplementary Material

Online Supplementary Material
