# Supplementary material for: Healthcare Resource Utilization and Costs Related to Falls and Fractures Among People With Type 2 Diabetes Receiving Basal Insulin: The FRAGILE Study
Source: J Health Econ Outcomes Res. 2025 Apr 28;12(1):171–83. doi: 10.36469/001c.133274 (PMC12043012; doi:10.36469/001c.133274)
Supplement: Online Supplementary Material [file jheor_2025_12_1_133274_280639.pdf]

Journal of Health Economics and Outcomes Research

Online Supplementary Material

Healthcare Resource Utilization and Costs Related to Falls and Fractures Among People With Type 2 Diabetes Receiving Basal Insulin: The FRAGILE Study. *JHEOR*. 2025;12(1):171-183. doi:10.36469/jheor.2025.133274

Table S1: Study Attrition

Table S2: Demographic and Baseline Characteristics of Cohorts in Subgroup 65 Years and Older Before and After Propensity Score Matching

Table S3: Fall/Fracture-Related Costs After Propensity Score Matching in the 65 Years and Older Subpopulation

Figure S1: Fall/Fracture-Related Healthcare Resource Utilization in People With Type 2 Diabetes 65 Years and Older

Figure S2: Association Between Fall/Fracture and Hypoglycemia in People With Type 2 Diabetes 65 Years and Older

This supplementary material has been provided by the authors to give readers additional information about their work.

This is an open-access article distributed under the terms of the Creative Commons Attribution 4.0 International License (CCBY-4.0). View this license's legal deed at http://creativecommons.org/licenses/by/4.0 and legal code at http://creativecommons.org/licenses/by/4.0/legalcode for more information.

BACKGROUND AND METHODS

As many organizations define older adults as those who are aged 65 years and older[1-4] and given that the incidence of fractures increases with age after 65 years,[5] the primary analysis was repeated in this subgroup. Demographic and baseline characteristics before and after PSM are detailed in Table S2. For the subgroup aged 65 years and older, the basal insulin–naive model (n = 10 350/group) was adjusted for region, while the basal insulin–switch model (n = 4939/group) was adjusted for healthcare plan type, region, and number of oral antidiabetic drugs.

RESULTS AND DISCUSSION

Results showed numerically higher rates of fall/fractures, as well as numerically higher rates of overall healthcare resource utilization in people aged 65 years and older compared with those aged 50 years and older. These results are consistent with incidence reports[5,6] and with findings of a study in US veterans demonstrating that individuals with vs without hypoglycemia had increased emergency department visits, hospital admissions, and long-term care placements.[7]

Healthcare resource utilization patterns according to use of Gla-300 or long-acting basal insulins/NPH in older adults were consistent with those in the population aged 50 years and older, with numerically lower hospitalization and emergency department visit event rates in adults aged 65 years and older who initiated Gla-300 vs long-acting basal insulins/NPH and in those who switched to Gla-300 vs long-acting basal insulins/NPH (**Figure S1**). Again, consistent with the population aged 50 years and older, and as expected, fall/fracture event rates in older adults were numerically higher for people with hypoglycemia vs those without hypoglycemia, regardless of whether they were newly initiating basal insulin or switching basal insulin (**Figure S2**). In contrast to findings in the overall basal insulin–naive group (aged 50 years and older), in insulin-naive older adults aged 65 years and older, in those newly initiating basal insulin treatment, fall/fracture event rates were lower with long-acting basal insulins/NPH vs Gla-300. However, it is notable that despite this, hospitalization and emergency department visit event rates remained lower for Gla-300 than for long-acting basal insulins/NPH in this subgroup. It is also notable that when initiating treatment with long-acting basal insulin/NPH, the fall/fracture event rate was lower in the 65 years and older cohort than in the 50 years and older cohort, which could suggest that factors not accounted for by propensity score matching could have contributed to this outcome. The association between falls and fractures and hypoglycemia in older adults was greatest in those who switched to long-acting basal insulins/NPH, as evidenced by the very high event rates in people with hypoglycemia. In contrast, rates were substantially lower in those who switched to Gla-300 and were in fact similar to rates observed in the 50 years and older subgroup.

The pattern of fall/fracture-related costs in older adults was largely similar to that of the overall population. In the basal insulin–naive cohort, all costs were numerically higher for Gla-300 vs long-acting basal insulins/NPH, and in the basal insulin–switch cohort, costs were substantially lower in those who switched to Gla-300 vs long-acting basal insulins/NPH, with the reduction in total healthcare costs for Gla-300 being driven by lower medical costs (as pharmacy costs were numerically higher for Gla-300). Hospitalization costs were also substantially lower for people switching to Gla-300 vs long-acting basal insulins/NPH, and emergency department visit costs were also lower for Gla-300, although the cost difference was not as high. Adjusted cost ratios are listed in **Table S3**. It is notable that the numerically higher hospitalization costs for older adults initiating basal insulin therapy with Gla-300 vs long-acting basal insulins/NPH occurred despite numerically lower HCRU for Gla-300 vs long-acting basal insulins/NPH. These increased costs could be due to the higher fall/fracture rate in older adults and the likely increased cost associated with medical care in this population.

REFERENCES

1. Institute of Medicine (US) Committee to Design a Strategy for Quality Review and Assurance in Medicare; Lohr KN, ed. Washington (DC): Medicare: A Strategy for Quality Assurance: Volume 1. The Elderly Population. Accessed February 2025. https://www.ncbi.nlm.nih.gov/books/NBK235450/

2. National Council on Aging. Aging in America: Get the facts on older Americans. Accessed February 2025. https://www.ncoa.org/article/get-the-facts-on-older-americans/

3. Organisation for Economic Co-operation and Development. Elderly population. Accessed February 2025. https://www.oecd.org/en/data/indicators/elderly-population.html

4. United Nations International Day of Older Persons 1 October: 2024 Theme: Ageing with Dignity: The Importance of Strengthening Care and Support Systems for Older Persons Worldwide. Accessed February 2025. https://www.un.org/en/observances/older-persons-day

5. Bergh C, Wennergren D, Moller M, Brisby H. Fracture incidence in adults in relation to age and gender: a study of 27,169 fractures in the Swedish Fracture Register in a well-defined catchment area. *PLoS One*. 2020;15(12):e0244291. doi:10.1371/journal.pone.0244291

6. Centers for Disease Control and Prevention. About older adult fall prevention. Updated January 17, 2024. Accessed June 11, 2024. https://www.cdc.gov/falls

7. Zhao Y, Kachroo S, Kawabata H, et al. Association between hypoglycemia and fall-related fractures and health care utilization in older veterans with type 2 diabetes. *Endocr Pract*. 2016;22(2):196-204. doi:10.4158/EP15640.OR

Table S1. Study Attrition

	Basal Insulin–Naive Cohort				Basal Insulin–Switch Cohort			
	Gla-300		Long-Acting Basal Insulin/NPH		Gla-300		Long-Acting Basal Insulin/NPH	
	No. Included	Percentage of Previous Excluded	No. Included	Percentage of Previous Excluded	No. Included	Percentage of Previous Excluded	No. Included	Percentage of Previous Excluded
Inclusion criteria								
Had ≥1 prescription claim for basal insulin (Gla-300 group: Gla-300; long-acting basal insulin group: Gla-100 or insulin detemir) during identification period (April 1, 2015–April 30, 2021)	95 564		838 996		95 564		838 996	
Had ≥1 prescription claim for basal insulin (NPH, Gla-300, or insulin detemir different from index basal insulin]) during the baseline period. If patient had ≥1 type of basal insulin treatment during the baseline period, the basal insulin treatment closest to switch will be chosen. No fills of multiple basal insulin therapies on index date.					28 431	29.8	42 460	5.1
Were ≥50 years of age on index date	84 485	88.4	713 965	85.1	25 090	88.2	35 563	83.8
Had continuous medical and prescription drug coverage for 180 days prior to index date (baseline period)	51 804	61.3	355 300	49.8	21 526	85.8	29 015	81.6
Exclusion criteria								
Patients with any diagnosis of type 1 diabetes (ICD-9-CM codes 250.x1 or 250.x3, ICD-10-CM code E10) or secondary diabetes (ICD-9-CM code 249.xx, ICD-10-CM code E08 or E09) at any time during the study period	30 207	58.3	213 322	60.0	11 238	52.2	15 488	53.4
Patients with any basal insulin therapy (eg, Gla-300, other long-acting basal insulins) during the baseline period	18 905	62.6	145 969	68.4				
Patients with index therapy during the baseline period					11 238	100	14 399	93.0
Patients with any insulin degludec at any time during the study period	14 534	76.9	137 332	94.1	10 066	89.6	13 608	94.5
Analysis population	14 534		137 332		10 066		13 608	

Abbreviations: Gla-100, insulin glargine 100 U/mL; Gla-300, insulin glargine 300 U/mL; ICD-9-CM, *International Classification of Diseases, Ninth Revision, Clinical Modification*; ICD-10-CM, *International Classification of Diseases, Tenth Revision, Clinical Modification*; NPH, neutral protamine Hagedorn.

Table S2. Demographic and Baseline Characteristics of Cohorts in Subgroup 65 Years and Older Before and After PSM

**Demographic and Clinical Characteristics**	**Basal Insulin–Naive Population**						**Basal Insulin–Switch Population**					
	**Before PSM**			**After PSM**			**Before PSM**			**After PSM**		
	**Gla-300 (n = 10 351)**	**Other Long-Acting BIs (n = 84 925)**	**SMD**	**Gla-300 (n = 10 350)**	**Other Long-Acting BIs (n = 10 350)**	**SMD**	**Gla-300 (n = 5877)**	**Other Long-Acting BIs (n = 7004)**	**SMD**	**Gla-300 (n = 4939)**	**Other Long-Acting BIs (n = 4939)**	**SMD**
Age (years), continuous												
Mean (SD)	73.0 (5.9)	74.0 (6.7)	-0.16	73.0 (5.9)	73. (6.1)	0.00	72.5 (5.8)	73.8 (6.8)	-0.21	72.6 (5.9)	72.8 (6.3)	-0.03
Median (IQR)	72.0 (68.0-77.0)	73.0 (68.0-79.0)	N/A	72.0 (68.0-77.0)	72.0 (68.0-77.0)	N/A	71.0 (68.0-76.0)	72.0 (68.0-78.0)	N/A	71.0 (68.0-76.0)	71.0 (68.0-77.0)	N/A
Minimum (maximum)	65.0 (90.0)	65.0 (90.0)	N/A	65.0 (90.0)	65.0 (90.0)	N/A	65.0 (90.0)	65.0 (90.0)	N/A	65.0 (90.0)	65.0 (90.0)	N/A
Gender, n (%)												
Male	5144 (49.7)	42 421 (50.0)	0.01	5144 (49.7)	5174 (50.0)	0.01	2818 (47.9)	3073 (43.9)	0.08	2329 (47.2)	2252 (45.6)	0.03
Female	5207 (50.3)	42 504 (50.0)	0.01	5206 (50.3)	5176 (50.0)	0.01	3059 (52.1)	3931 (56.1)	0.08	2610 (52.8)	2687 (54.4)	0.03
Health plan type, n (%)												
Commercial	361 (3.5)	6160 (7.3)	0.17	361 (3.5)	342 (3.3)	0.01	632 (10.8)	1118 (16.0)	0.15	607 (12.3)	785 (15.9)	0.11
Medicare	9986 (96.5)	78 738 (92.7)	0.17	9985 (96.5)	10 003 (96.6)	0.01	5243 (89.2)	5884 (84.0)	0.15	4331 (87.7)	4152 (84.1)	0.10
Region, n (%)												
Northeast	861 (8.3)	9107 (10.7)	0.56	861 (8.3)	957 (9.2)	0.47	573 (9.7)	799 (11.4)	0.19	478 (9.7)	523 (10.6)	0.14
Midwest	1273 (12.3)	16 083 (18.9)	0.47	1273 (12.3)	1680 (16.2)	0.41	899 (15.3)	1331 (19.0)	0.13	788 (16.0)	865 (17.5)	0.13
South	6214 (60.0)	39 278 (46.3)	0.20	6213 (60.0)	5103 (49.3)	0.16	3168 (53.9)	3462 (49.4)	0.12	2666 (54.0)	2486 (50.3)	0.05
West	1994 (19.3)	20 350 (24.0)	0.49	1994 (19.3)	2593 (25.1)	0.34	1229 (20.9)	1403 (20.0)	0.21	1002 (20.3)	1057 (21.4)	0.13
Other/unknown	9 (0.1)	107 (0.1)	0.59	9 (0.1)	17 (0.2)	0.49	8 (0.1)	9 (0.1)	0.21	5 (0.1)	8 (0.2)	0.15
Index basal insulin, n (%)												
Gla-100		62 989 (74.2)			7658 (74.0)			6315 (90.2)			4471 (90.5)	
IDet	N/A	16 069 (18.9)	N/A	N/A	1895 (18.3)	N/A	N/A	633 (9.0)	N/A	N/A	421 (8.5)	N/A
NPH		5867 (6.9)			797 (7.7)			56 (0.8)			47 (1.0)	
Baseline comorbidities and complications, n (%)												
Anemia	1962 (19.0)	21 582 (25.4)	-0.16	1962 (19.0)	1910 (18.5)	0.01	1074 (18.3)	2027 (28.9)	-0.25	955 (19.3)	970 (19.6)	-0.01
Hypertension	8513 (82.2)	70 675 (83.2)	-0.03	8512 (82.2)	8463 (81.8)	0.01	4956 (84.3)	6080 (86.8)	-0.07	4159 (84.2)	4123 (83.5)	0.02
Hyperlipidemia	6948 (67.1)	51 631 (60.8)	0.13	6947 (67.1)	6927 (66.9)	0.00	3935 (67.0)	4559 (65.1)	0.04	3291 (66.6)	3106 (62.9)	0.08
Diabetic neuropathy	3140 (30.3)	25 980 (30.6)	-0.01	3139 (30.3)	3074 (29.7)	0.01	2152 (36.6)	2531 (36.1)	0.01	1789 (36.2)	1668 (33.8)	0.05
Diabetic nephropathy	1001 (9.7)	8632 (10.2)	-0.02	1001 (9.7)	971 (9.4)	0.01	710 (12.1)	852 (12.2)	0.00	595 (12.0)	558 (11.3)	0.02
Diabetic retinopathy	1181 (11.4)	8302 (9.8)	0.05	1181 (11.4)	1173 (11.3)	0.00	974 (16.6)	1047 (14.9)	0.04	809 (16.4)	756 (15.3)	0.03
Overweight/obesity	2068 (20.0)	18 037 (21.2)	-0.03	2068 (20.0)	2015 (19.5)	0.01	1410 (24.0)	1787 (25.5)	-0.04	1179 (23.9)	1125 (22.8)	0.03
Chronic kidney disease	3344 (32.3)	28 685 (33.8)	-0.03	3343 (32.3)	3278 (31.7)	0.01	1927 (32.8)	2705 (38.6)	-0.12	1637 (33.1)	1588 (32.2)	0.02

Table S2. Demographic and Baseline Characteristics of Cohorts in Subgroup 65 Years and Older Before and After PSM

Demographic and Clinical Characteristics	Basal Insulin–Naive Population						Basal Insulin–Switch Population					
	Before PSM			After PSM			Before PSM			After PSM		
	Gla-300 (n = 10 351)	Other Long-Acting BIs (n = 84 925)	SMD	Gla-300 (n = 10 350)	Other Long-Acting BIs (n = 10 350)	SMD	Gla-300 (n = 5877)	Other Long-Acting BIs (n = 7004)	SMD	Gla-300 (n = 4939)	Other Long-Acting BIs (n = 4939)	SMD
Depression	1427 (13.8)	14 526 (17.1)	-0.09	1427 (13.8)	1412 (13.6)	0.00	822 (14.0)	1531 (21.9)	-0.21	728 (14.7)	756 (15.3)	-0.02
Anxiety	885 (8.5)	9913 (11.7)	-0.10	885 (8.6)	881 (8.5)	0.00	525 (8.9)	996 (14.2)	-0.17	474 (9.6)	488 (9.9)	-0.01
Ischemic heart disease	2698 (26.1)	26 529 (31.2)	-0.11	2698 (26.1)	2625 (25.4)	0.02	1664 (28.3)	2403 (34.3)	-0.13	1424 (28.8)	1388 (28.1)	0.02
Myocardial infarction	192 (1.9)	4145 (4.9)	-0.17	192 (1.9)	184 (1.8)	0.01	116 (2.0)	411 (5.9)	-0.20	115 (2.3)	108 (2.2)	0.01
Transient ischemic attack	159 (1.5)	2281 (2.7)	-0.08	159 (1.5)	157 (1.5)	0.00	100 (1.7)	222 (3.2)	-0.10	86 (1.7)	86 (1.7)	0.00
Stroke	330 (3.2)	5625 (6.6)	-0.16	330 (3.2)	334 (3.2)	0.00	190 (3.2)	611 (8.7)	-0.23	177 (3.6)	198 (4.0)	-0.02
Peripheral arterial disease	505 (4.9)	4273 (5.0)	-0.01	504 (4.9)	493 (4.8)	0.00	261 (4.4)	465 (6.6)	-0.10	228 (4.6)	242 (4.9)	-0.01
Heart failure	1422 (13.7)	18 462 (21.7)	-0.21	1422 (13.7)	1324 (12.8)	0.03	921 (15.7)	1832 (26.2)	-0.26	814 (16.5)	860 (17.4)	-0.02
Baseline oral and injectable antidiabetics, n (%)												
Diabetes polypharmacy[a]	330 (3.2)	2406 (2.8)	0.02	330 (3.2)	332 (3.2)	0.00	120 (2.0)	99 (1.4)	0.05	91 (1.8)	85 (1.7)	0.01
Prior insulin, n (%)[b]												
Switched from Gla-100							3952 (67.2)	159 (2.3)	N/A	3343 (67.7)	113 (2.3)	N/A
Switched from IDet	N/A	N/A	N/A	N/A	N/A	N/A	1493 (25.4)	4 336 (61.9)	N/A	1245 (25.2)	3057 (61.9)	N/A
Switched from NPH							432 (7.4)	2 509 (35.8)	N/A	351 (7.1)	1769 (35.8)	N/A
Biguanides	5094 (49.2)	39 340 (46.3)	0.06	5094 (49.2)	5242 (50.6)	-0.03	2597 (44.2)	2 936 (41.9)	0.05	2206 (44.7)	2220 (44.9)	-0.01
Sulfonylureas	4311 (41.6)	33 249 (39.2)	0.05	4311 (41.7)	4441 (42.9)	-0.03	1487 (25.3)	1 643 (23.5)	0.04	1241 (25.1)	1215 (24.6)	0.01
Thiazolidindiones	907 (8.8)	5727 (6.7)	0.08	907 (8.8)	937 (9.1)	-0.01	299 (5.1)	293 (4.2)	0.04	244 (4.9)	226 (4.6)	0.02
DPP-4 inhibitors	1848 (17.9)	13 123 (15.5)	0.06	1848 (17.9)	1881 (18.2)	-0.01	728 (12.4)	732 (10.5)	0.06	596 (12.1)	561 (11.4)	0.02
SGLT2 inhibitors	1036 (10.0)	4883 (5.7)	0.16	1035 (10.0)	981 (9.5)	0.02	542 (9.2)	363 (5.2)	0.16	426 (8.6)	335 (6.8)	0.07
GLP-1 RA	1465 (14.2)	6723 (7.9)	0.20	1464 (14.1)	1412 (13.6)	0.01	965 (16.4)	639 (9.1)	0.22	745 (15.1)	590 (11.9)	0.09
α-Glucosidase inhibitors	67 (0.6)	480 (0.6)	0.01	67 (0.6)	66 (0.6)	0.00	24 (0.4)	22 (0.3)	0.02	21 (0.4)	17 (0.3)	0.01
Other hypoglycemic agents	194 (1.9)	1166 (1.4)	0.04	193 (1.9)	196 (1.9)	0.00	80 (1.4)	74 (1.1)	0.03	66 (1.3)	61 (1.2)	0.01
Combination formulations	833 (8.0)	4028 (4.7)	0.14	832 (8.0)	772 (7.5)	0.02	387 (6.6)	267 (3.8)	0.13	292 (5.9)	246 (5.0)	0.04
History of hypoglycemia, n (%)												
Any hypoglycemia during baseline (yes/no)	338 (3.3)	4195 (4.9)	-0.08	338 (3.3)	340 (3.3)	0.00	293 (5.0)	784 (11.2)	-0.23	265 (5.4)	304 (6.2)	-0.03
History of fall/fractures, n (%)												
Any fall/fractures during the baseline (yes/no)	471 (4.6)	7549 (8.9)	-0.17	471 (4.6)	464 (4.5)	0.00	283 (4.8)	832 (11.9)	-0.26	263 (5.3)	281 (5.7)	-0.02

Table S2. Demographic and Baseline Characteristics of Cohorts in Subgroup 65 Years and Older Before and After PSM

Demographic and Clinical Characteristics	Basal Insulin–Naive Population						Basal Insulin–Switch Population					
	Before PSM			After PSM			Before PSM			After PSM		
	Gla-300 (n = 10 351)	Other Long-Acting BIs (n = 84 925)	SMD	Gla-300 (n = 10 350)	Other Long-Acting BIs (n = 10 350)	SMD	Gla-300 (n = 5877)	Other Long-Acting BIs (n = 7004)	SMD	Gla-300 (n = 4939)	Other Long-Acting BIs (n = 4939)	SMD
No. of OADs (continuous), n (%)												
Mean (SD)	1.52 (1.11)	1.28 (1.09)	0.22	1.52 (1.11)	1.54 (1.15)	-0.02	1.21 (0.99)	1.00 (0.96)	0.22	1.18 (0.97)	1.11 (0.98)	0.08
Median (IQR)	1.0 (1.0-2.0)	1.0 (0.0-2.0)	N/A	1.0 (1.0-2.0)	1.0 (1.0-2.0)	N/A	1.0 (0.0-2.0)	1.0 (0.0-2.0)	N/A	1.0(0.0-2.0)	1.0 (0.0-2.0)	N/A
Minimum (maximum)	0.0 (6.0)	0.0 (7.0)	N/A	0.0 (6.0)	0.0 (6.0)	N/A	0.0 (6.0)	0.0 (5.0)	N/A	0.0 (6.0)	0.0 (5.0)	N/A
No. of OADs (categorical), n (%)												
0	2100 (20.3)	24 692 (29.1)	0.16	2100 (20.3)	2277 (22.0)	0.09	1565 (26.6)	2554 (36.5)	0.15	1342 (27.2)	1554 (31.5)	0.06
1	3182 (30.7)	26 203 (30.9)	0.31	3182 (30.7)	2899 (28.0)	0.07	2211 (37.6)	2514 (35.9)	0.33	1879 (38.0)	1809 (36.6)	0.14
2	3123 (30.2)	22 195 (26.1)	0.28	3123 (30.2)	3059 (29.6)	0.10	1524 (25.9)	1437 (20.5)	0.30	1274 (25.8)	1143 (23.1)	0.13
≥3	1946 (18.8)	11 835 (13.9)	0.27	1945 (18.8)	2115 (20.4)	0.09	577 (9.8)	499 (7.1)	0.33	444 (9.0)	433 (8.8)	0.15
Risk factors for falls and fractures												
Frailty and age-related factors, n (%)												
Vision loss and impairment[c]	97 (0.9)	1097 (1.3)	-0.03	97 (0.9)	105 (1.0)	-0.01	56 (1.0)	137 (2.0)	-0.08	52 (1.1)	55 (1.1)	-0.01
Cognitive impairment and dementia	458 (4.4)	9375 (11.0)	-0.25	458 (4.4)	424 (4.1)	0.02	311 (5.3)	1091 (15.6)	-0.34	299 (6.1)	338 (6.8)	-0.03
Age-related physical debility	35 (0.3)	951 (1.1)	-0.09	35 (0.3)	33 (0.3)	0.00	23 (0.4)	119 (1.7)	-0.13	22 (0.4)	21 (0.4)	0.00
Osteoarthritis	1941 (18.8)	18 642 (22.0)	-0.08	1941 (18.8)	1885 (18.2)	0.01	1157 (19.7)	1 774 (25.3)	-0.14	1012 (20.5)	1013 (20.5)	0.00
Osteoporosis	509 (4.9)	4131 (4.9)	0.00	509 (4.9)	495 (4.8)	0.01	258 (4.4)	397 (5.7)	-0.06	222 (4.5)	206 (4.2)	0.02
Medications, n (%)												
Antipsychotics	286 (2.8)	3942 (4.6)	-0.10	286 (2.8)	285 (2.8)	0.00	175 (3.0)	415 (5.9)	-0.14	163 (3.3)	177 (3.6)	-0.02
Antidepressants	2838 (27.4)	24 025 (28.3)	-0.02	2838 (27.4)	2774 (26.8)	0.01	1808 (30.8)	2469 (35.3)	-0.10	1545 (31.3)	1536 (31.1)	0.00
Benzodiazepines	124 (1.2)	864 (1.0)	0.02	124 (1.2)	123 (1.2)	0.00	72 (1.2)	67 (1.0)	0.03	54 (1.1)	49 (1.0)	0.01
Polypharmacy[d]	4085 (39.5)	34 168 (40.2)	-0.02	4085 (39.5)	3989 (38.5)	0.02	2893 (49.2)	3567 (50.9)	-0.03	2449 (49.6)	2425 (49.1)	0.01
High-risk triggering events and conditions, n (%)												
Hypotension and postural hypotension	304 (2.9)	5579 (6.6)	-0.17	304 (2.9)	279 (2.7)	0.01	156 (2.7)	533 (7.6)	-0.23	148 (3.0)	147 (3.0)	0.00
Vertigo	121 (1.2)	817 (1.0)	0.02	121 (1.2)	125 (1.2)	0.00	55 (0.9)	75 (1.1)	-0.01	46 (0.9)	49 (1.0)	-0.01
Syncope and collapse	326 (3.1)	4 394 (5.2)	-0.10	326 (3.1)	302 (2.9)	0.01	180 (3.1)	447 (6.4)	-0.16	164 (3.3)	188 (3.8)	-0.03
Nonepileptic seizure	75 (0.7)	1765 (2.1)	-0.12	75 (0.7)	64 (0.6)	0.01	58 (1.0)	207 (3.0)	-0.14	55 (1.1)	55 (1.1)	0.00
Malaise and fatigue	1693 (16.4)	20 830 (24.5)	-0.20	1693 (16.4)	1603 (15.5)	0.02	877 (14.9)	1941 (27.7)	-0.32	784 (15.9)	809 (16.4)	-0.01

Table S2. Demographic and Baseline Characteristics of Cohorts in Subgroup 65 Years and Older Before and After PSM

Demographic and Clinical Characteristics	Basal Insulin–Naive Population						Basal Insulin–Switch Population					
	Before PSM			After PSM			Before PSM			After PSM		
	Gla-300 (n = 10 351)	Other Long-Acting BIs (n = 84 925)	SMD	Gla-300 (n = 10 350)	Other Long-Acting BIs (n = 10 350)	SMD	Gla-300 (n = 5877)	Other Long-Acting BIs (n = 7004)	SMD	Gla-300 (n = 4939)	Other Long-Acting BIs (n = 4939)	SMD
Healthcare utilization, n (%)												
≥1 inpatient admission	1315 (12.7)	28 253 (33.3)	-0.50	1315 (12.7)	1287 (12.4)	0.01	754 (12.8)	2581 (36.9)	-0.58	743 (15.0)	797 (16.1)	-0.03
≥1 ED visit	2367 (22.9)	33 454 (39.4)	-0.36	2367 (22.9)	2323 (22.4)	0.01	1334 (22.7)	2970 (42.4)	-0.43	1208 (24.5)	1293 (26.2)	-0.04

Abbreviations: DDP4, dipeptidyl peptidase 4; ED, emergency department; GLP-1 RA, glucagon-like peptide 1 receptor agonists; IDet, insulin detemir; Gla-100, insulin glargine 100 U/mL; Gla-300, insulin glargine 300 U/mL; IQR, interquartile range; N/A, not available; NPH, neutral protamine Hagedorn; OADs, oral antidiabetic drugs; PSM, propensity score–matching; SGLT2, sodium-glucose cotransporter-2; SMD, standardized mean difference.

[a]At least 3 different noninsulin antidiabetics within 30 days of index.

[b]SMD for prior insulin is N/A because prior insulin was not included as a covariate in the PSM model.

[c]Vision loss and impairment beyond diabetic ophthalmopathy (eg, cataracts).

[d]At least 5 different nondiabetes medications within 30 days of index.

Table 3. Fall/Fracture-Related Costs After PSM in the 65 Years and Older Subpopulation

	Basal Insulin–Naive Cohort (n = 10 350)				Basal Insulin–Switch Cohort (n = 4939)			
	Gla-300	Long-Acting BIs/NPH	Cost Difference (Gla-300 vs Long-Acting BIs /NPH)	Adjusted Cost Ratio (95% CI): Gla-300 vs Long-Acting BIs /NPH	Gla-300	Long-Acting BIs/NPH	Cost Difference (Gla-300 vs Long-Acting BIs /NPH)	Adjusted Cost Ratio (95% CI): Gla-300 vs Long-Acting BIs /NPH
Hospitalization	91 128 (n = 255)	82 202 (n = 262)	+8925	1.11 (0.70-1.77)	70 363 (n = 117)	116 655 (n = 140)	-46 292	0.48 (0.31-0.76)
ED visit	25 679 (n = 467)	24 427 (n = 474)	+1252	0.90 (0.76-1.07)	22 001 (n = 221)	24 571 (n = 262)	-2569	1.27 (1.04-1.55)
Total health care	131 651 (n = 670)	121 766 (n = 670)	+9885	0.73 (0.58-0.92)	103 492 (n = 309)	161 162 (n = 374)	-57 670	0.57 (0.42-0.77)
Medical	131 497	121 691	+9806	0.72 (0.58-0.90)	103 048	160 913	-57 865	0.58 (0.43-0.78
Pharmacy	153	74	+79	2.05 (0.74-5.65)	444	249	+195	5.10 (1.12-23.31)

Abbreviations: CI, confidence interval; ED, emergency department; Gla-300, insulin glargine 300 U/mL; NPH, neutral protamine Hagedorn; PSM, propensity score–matching.

Figure S1. Fall/Fracture-Related Healthcare Resource Utilization in People With Type 2 Diabetes Aged 65 Years and Older

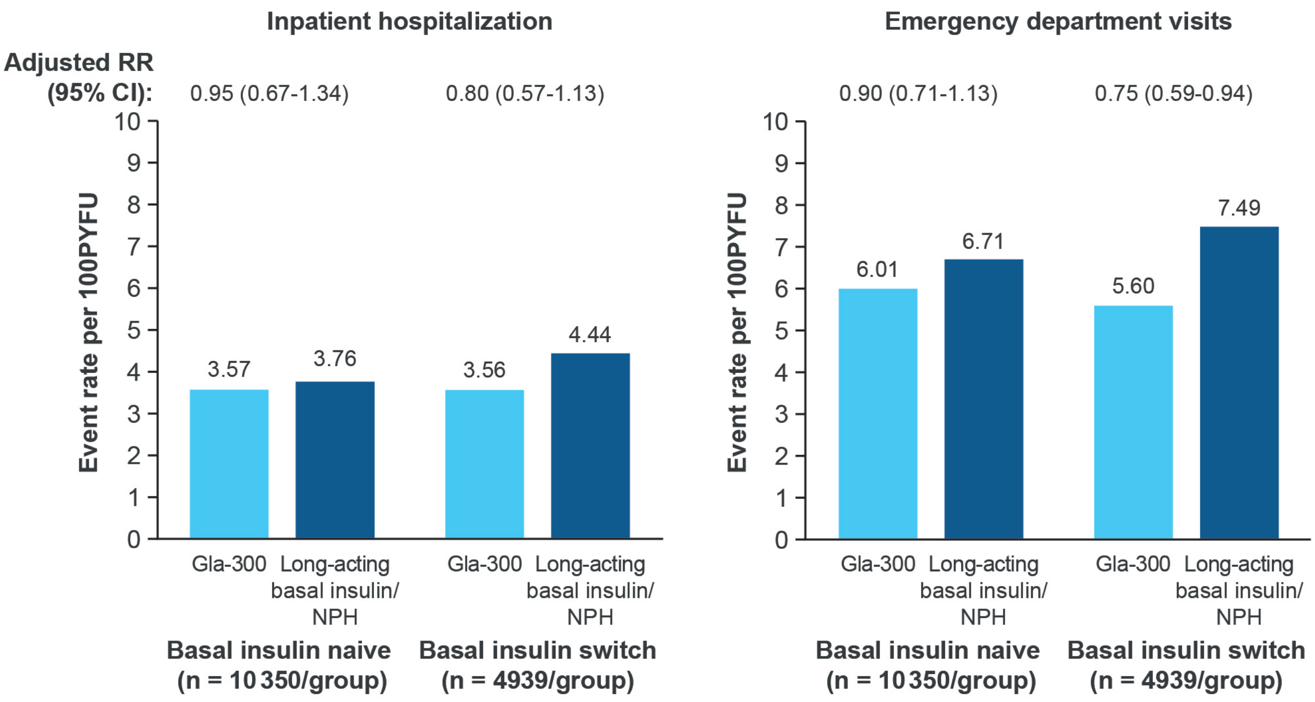

Abbreviations: CI, confidence interval; PYFU, person-years of follow-up; RR, rate ratio.

Figure S2. Association Between Fall/Fracture and Hypoglycemia in People With Type 2 Diabetes Aged 65 Years and Older

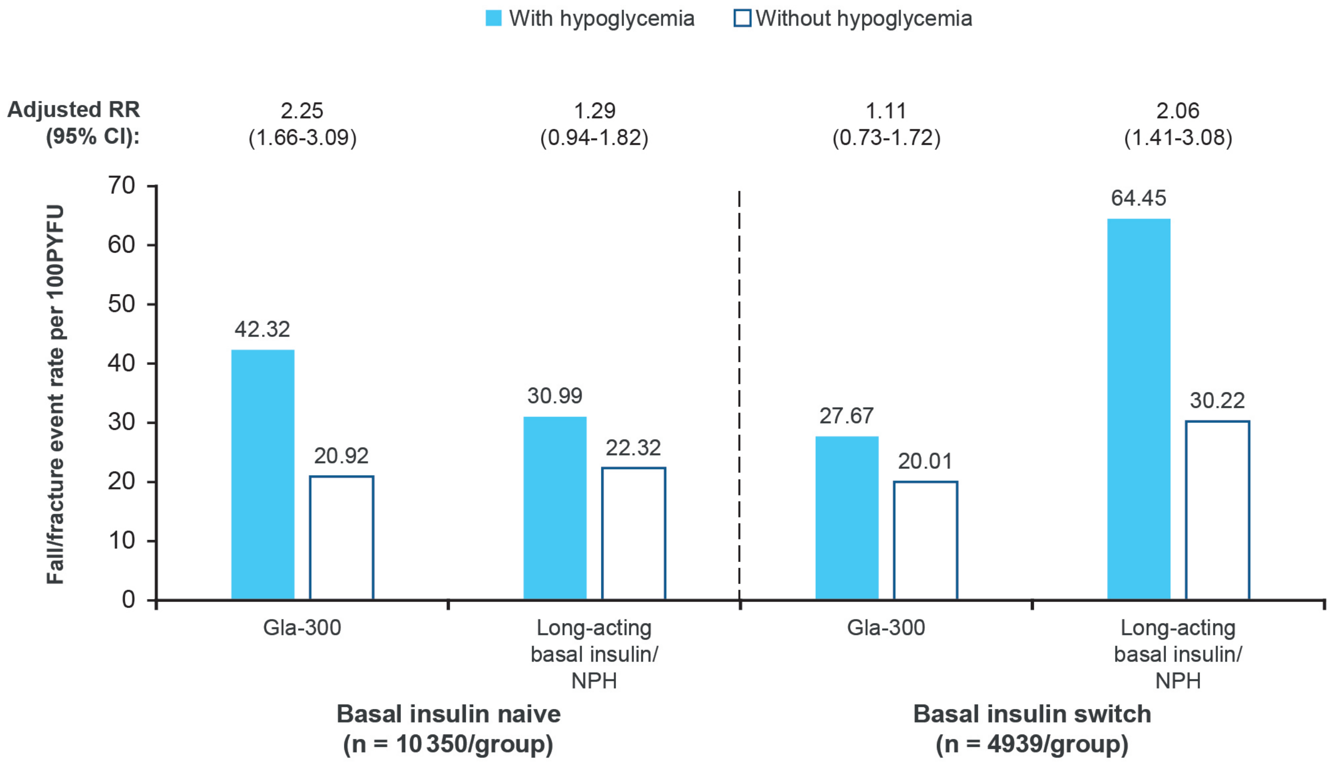

Abbreviations: CI, confidence interval; RR, rate ratio.
